# ERA5-based global assessment of irrigation requirement and validation

**DOI:** 10.1371/journal.pone.0250979

**Published:** 2021-04-30

**Authors:** Matteo Rolle, Stefania Tamea, Pierluigi Claps

**Affiliations:** Department of Environment, Land and Infrastructure Engineering, Politecnico di Torino, Torino, Italy; Soil and Water Resources Institute ELGO-DIMITRA, GREECE

## Abstract

While only 20% of harvested lands are actually irrigated, 40% of global agricultural production originates from irrigated areas. Therefore, assessing irrigation requirements is essential for the development of effective water-related policies for an efficient management of water resources. Moreover, global-scale analyses are becoming increasingly relevant, motivated by globalized production and international trade of food as well as by the need of common strategies to address climate change.

In this study, a comprehensive model to estimate crop growth and irrigation requirements of 26 main crops at global scale is presented. The model computes a soil water balance using daily precipitation and reference evapotranspiration based on a high-resolution ERA5 reanalysis dataset from the European Copernicus Program. The irrigation requirement, defined as the minimum water volume to avoid water stress, is computed for year 2000 at the resolution of 5 arc-min (or 0.0833°) and aggregated at different spatial and temporal scales for relevant analyses.

The estimated global irrigation requirements for 962 km^3^ is described in detail, also in relation to the spatial variability and to the monthly variation of the requirements. A focus on different areas of the world (California, Northern Italy and India) highlights the wealth of information provided by the model in different climatic conditions.

National data of irrigation withdrawals have been used for an extensive comparison with model results. A crop-specific validation has also been made for the State of California, comparing model results with local data of irrigation volume and independent estimates of crop water use. In both cases, we found a good agreement between model results and real data.

## 1. Introduction

Agriculture plays a main role in a world where population is expected to grow rapidly in the next decades. Agriculture is the human activity which requires most of the withdrawn freshwater, with 20% of irrigated harvested lands providing 40% of global food production [1]. Ensuring the availability of irrigation water to meet agricultural requirements is a primary concern for the future of humanity, due to the difficulty of finding a balance between the increasing needs for food production, threats due to climate change, and other human uses [2]. The climate change is expected to produce a large-scale impact on all the human activities and our mitigation strategies must match up the complexity of a globalized food production. Therefore, a global assessment of irrigation requirements is important to manage agricultural and water resources at different spatial scales, investigate crop shifts [3] and understand new opportunities of global food trade for water conservation [4].

Along this study, the irrigation requirement is intended as the amount of water provided to crops when precipitation does not entirely satisfy the evapotranspiration demand. A number of high-resolution assessments of global irrigation requirements exist. The first ones were based on WaterGAP [5], WATERSIM [6], LPJmL [7] and H07 [8] models, which mostly did not consider multi-cropping practices (i.e. the sequence of multiple growing seasons on the same area, e.g. “winter” and “spring” wheat) and provided little crop-specific results. The comparison in Table 1 shows that latest assessments are generally referred to year 2000, because of the availability of crop-specific information of irrigated areas for that year. This is the case for the GCWM [9], GEPIC [10], the assessment based on the CROPWAT model proposed by FAO [11], and the WATNEEDS model [12].

**Table 1:** Summary of models that estimate global irrigation requirement.

Global Models			Spatial resolution	Base year	MCP	Precipitation	Reference evapotranspiration	
2021	This study		0.0833°	2000	yes	0.25° (d)	0.25° (d)	HS
2020	[12]	WATNEEDS	0.0833°	2000^(a)^, 2016	yes	0.05° (d), 0.5° (d)*	0.5° (m)	PM
2011	[11]	CROPWAT (FAO)	0.0833°	2000^(b)^	no	0.5° (m)	0.166° (m-LTA)	PM
2010	[10]	GEPIC	0.5°	2000	yes	0.5° (m)	0.5° (m)	HS
2010	[9]	GCWM	0.0833°	2000^(a)^	yes	0.5° (m), 0.166° (m-LTA)**	0.5° (m), 0.166° (m-LTA)**	PT, PM
2008	[8]	H07	1°	1991^(c)^	no	1° (d)	1° (d)	SEB
2008	[7]	LPJmL	0.5°	1985^(d)^	no	0.5° (m)	0.5° (m)	PM
2007	[6]	WATERSIM (IWM)	0.1°	2000	no	0.5° (m)	0.5° (m)	PM
2002	[5]	WaterGAP	0.5°	1995	no	0.166° (m-LTA)	0.166° (m-LTA)	PT

Acronyms and notes used in the table. MCP: Multi-Cropping Practices. Reference Evapotranspiration methods: PM (Penman-Monteith), PT (Priestley-Taylor), HS (Hargreaves-Samani), SEB (Surface Energy Balance). Temporal resolution of data: (d) daily, (m) monthly, (m-LTA) monthly Long-Time Average (1961–1990).

^(^*^)^ 0.05° in the region between 50°N– 50°S, 0.5° in the rest of the world.

^(^**^)^ effective resolution of 0.166°, matching the two datasets.

^(a)^ Average result for the period 1998–2002.

^(b)^ Average result for the period 1996–2005.

^(c)^ The assessment provided an average result, using input data for the period 1986–1995.

^(d)^ The map of irrigated crops refers to year 2000. The assessment provides an average estimation using monthly climate data for the period 1971–2000.

Table 1 shows that the spatial resolution of global models has increased over the years, mainly because of the release of crop-specific datasets of irrigated areas. With the exception of Chiarelli et al. [12], who uses high-resolution daily precipitation, the previous models are rarely based on daily data or they provide results on lower spatial resolutions (e.g. 1° in Hanasaki et al. [8]). The present study introduces new daily global data for both precipitation and reference evapotranspiration, working on a high spatial resolution grid to best reproduce detail within the global scale. The reference evapotranspiration was calculated with temperature data, according to the Hargreaves-Samani method [13], and calibrated through global comparison with a widely used monthly dataset.

The introduction of hydroclimatic data from remote sensing has brought an important improvement to global models. Satellites are increasingly designed and used for agricultural applications [14], helping to identify irrigated lands [15] and indirectly to estimate irrigation volume [16]. The European Copernicus Program, started in 2014 as a continuation of GMES (Global Monitoring for Environment and Security), developed a system of satellites known as Sentinel Constellation to continuously monitor the Earth environment [17].

The model presented in this study aims to assess global irrigation requirements exploiting the potential of satellite products from the Copernicus Climate Change Service (C3S), in the computation of a soil-water balance approach based on the FAO methodology [18]. Model application is limited to year 2000, in order to match available data of crop harvested areas and growing periods. For year 2000, spatial coverage resulting from 26 main crops is provided by the MIRCA2000 database. Our estimate of irrigation requirements has been compared both to results of other models and to actual data from irrigation volumes on different spatial scales.

## 2. Data

The assessment of irrigation requirements is based on data of agricultural areas equipped for irrigation (*AEI*), and related calendars of growing periods. We used data from MIRCA2000 [19] which provides global gridded data on a 0.0833° spatial resolution (cell, or pixel, dimension of about 9x9 km at the Equator) for 26 different crops. The dataset includes information about cropping intensity, i.e. the ratio between gross harvested area (hectares that are actually harvested in one year, considering multiple growing seasons) and net sown area (area of the agricultural fields). Growing periods are defined for temporary crops (i.e. those that are both sown and harvested during the same year) on a monthly basis. In order to use this information within a daily assessment, we assumed that sowing and harvest occur on the 16th and the 15th day, respectively, of the given months, similarly to other studies (e.g. [20]). The length of a growing season is then considered as the number of days between the sowing and harvest dates. Growing periods for perennial crops (i.e. those that don’t need to be replanted every year, like fruit trees) are defined by the green-up dates instead. Such dates are taken from Chapagain & Hoekstra (2004) [21] and refer to the FAO agro-climatic zones system (GAEZ) [22].

### 2.1. Climate data

In this study we use the new global re-analysis dataset ERA5, released by Copernicus in 2018 [23], which includes hourly climate data that combines satellite information and ground measurements for 1950-present at the spatial resolution of 0.25° (i.e. about 28 km at the Equator). ERA5 is a global reanalysis product, based on a large number of hourly climate data sets, produced by the European Centre for Medium-Range Weather Forecasts (ECMWF) as an improvement over the previous ERA-Interim product (45 arc min, 6-hour temporal resolution). Climate data were downloaded through the Application Programming Interface (API) of ECMWF and subsequently processed to match the MIRCA2000 spatial grid.

The two main climate variables used in this study are precipitation (*P*) and reference evapotranspiration (*ET*_*0*_). In order to obtain daily rainfall, hourly precipitation available in ERA5 was aggregated summing data from 1:00h to 0:00h in each day. The reference evapotranspiration (*ET*_*0*_), defined as the evapotranspiration rate [mm/day] from a hypothetical well-watered grass surface with fixed characteristics [18],was calculated using the Hargreaves-Samani method [13]. The expression for daily ET_0_ [mm/day] reads:

$$ET_{0,i}=k_{HS}\times R_{a,i}\times \left(T_{mean,i}+17.8\right)\sqrt{T_{\text{max},i}-T_{\text{min},i}},$$ (1)

where *i* is the specific day, *T*_*max*,*i*_, *T*_*min*,*i*_ and *T*_*mean*,*i*_ are respectively the maximum, minimum and mean daily temperatures [°C] and *R*_*a*,*i*_ is the equivalent evaporation [mm], obtained by dividing the top-of-atmosphere radiation by the latent heat of vaporization of water (1/λ = 0.408). The empirical coefficient *k*_*HS*_ was initially fixed to 0.0023, as in the original formula proposed by Hargreaves-Samani [13]. All the variables required for the application of (1) were taken from ERA5 and were aggregated on a daily scale. The top-of-atmosphere radiation could be calculated using a geometric approach, but we choose to use the ERA5 product to be consistent with the spatial grid of temperature data.

Although the Hargreaves-Samani (HS) method is a valid alternative to the mostly recommended Penman-Monteith (PM) method [18], we introduce a spatial variability of the *k*_*HS*_ coefficient, that is calibrated to provide consistency with the annual estimates from PM available in the CRU Time-Series global dataset [24]. This procedure was developed according to the monthly calibration described by Heydari et al. [25]. The yearly HS evapotranspiration has been upscaled on a 0.5° resolution to match the spatial grid of CRU. The *k*_*HS*_ coefficient were obtained by multiplying 0.0023 by the ratios between annual PM and HS reference evapotranspiration values. Finally, the *k*_*HS*_ values were downscaled to match the MIRCA2000 grid, proportionally to the uncalibrated *ET*_*0*_ values in each pixel. The calibrated empirical coefficients were used to calculate the daily *ET*_*0*_, according to (1).

### 2.2. Analysis of reference evapotranspiration

The daily *ET*_*0*_ from Eq (1) has been computed on the global grid of 0.0833° for the whole year 2000. The mean value of the calibrated *k*_*HS*_ coefficients in Eq (1) was 0.0024 over the irrigated cells, with a standard deviation of 0.0004. The calibration produced higher coefficients along coastal and arid regions, which is consistent with the tendency of HS to underestimate *ET*_*0*_ in these scenarios [26].

**Fig 1:**
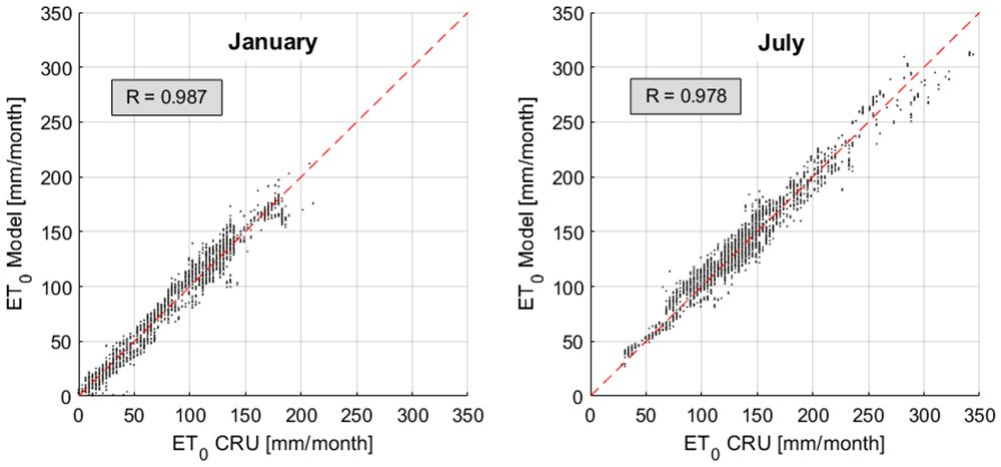
Comparison between the CRU-TS v.4 ET_0_ (Penman-Monteith, monthly data) and the monthly average ERA5-based Hargreaves-Samani ET_0_. The comparison was made considering all the cells around the world where the harvested area is at least 90% of the pixel, for the months of January (a) and July (b).

Results obtained have been compared with the PM reference evapotranspiration from CRU Time-Series [24] for the months of January and July, as shown in Fig 1. The scatter plots compare, on a global scale, the monthly *ET*_*0*_ from the PM and HS methods, in primarily cultivated areas, i.e. in cells where the harvested portion is greater than 90% of the pixel’s area. The Pearson correlation index (R) between the two datasets is 0.987 for January (Fig 1a) and 0.978 for July (Fig 1b). Similar results were obtained considering all the pixels with at least 1% of harvested area. This ensures that no substantial bias emerges from the monthly comparison.

**Fig 2:**
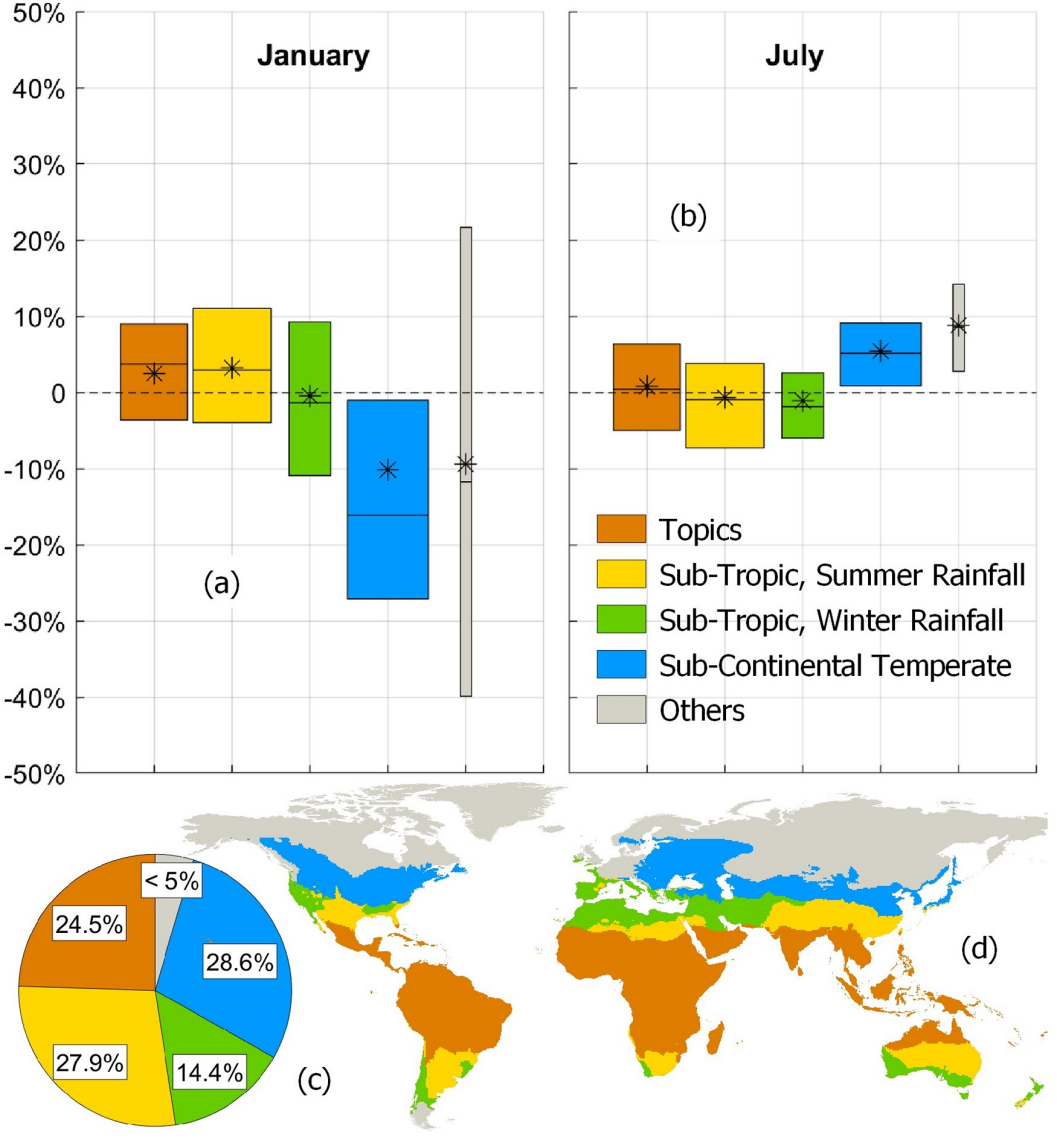
Boxplots and area-weighted means of percentage differences between monthly *ET*_*0*_ (Computed HS and PM from CRU-TS). Boxplots show values corresponding to 25%, 50% and 75% of harvested lands for six climate zones: Tropics (orange), Sub-tropics summer rainfall (yellow), Sub-tropics winter rainfall (green), Temperate sub-continental (blue). The grey boxplot describes the *ET*_*0*_ alignment over the least irrigated zones: Oceanic Temperate, Continental Temperate, Oceanic Boreal, Sub-Continental Boreal, Continental Boreal, Arctic. The horizontal dimension of the boxplot is proportional to the percentages of irrigated areas per climatic region, reported in the pie chart (c). Asterisks: area-weighted means of annual percentage differences. Fig 2d shows the GAEZ global agro-climatic classification of surface lands.

The two *ET*_*0*_ from HS and PM have also been compared in every cell, grouping the results by climate conditions. Fig 2 shows the boxplots over agro-climatic zones for the months of January (a) and July (b), according to the GAEZ thermal agro-classification [22]. Zones are obtained on the basis of climate data from CRU-TS [24] according to the indications given by Van Velthuizen et al. [27], and are shown in Fig 2d. The three levels in each boxplot show the percentage differences corresponding to 25%, 50% and 75% of harvested areas in each climatic zone. The asterisks indicate the area-weighted mean percentage difference. The pie chart in Fig 2c shows the percentage of irrigated areas per climate region: tropics (24.5%), sub-tropics summer rainfall (27.9%), sub-tropics winter rainfall (14.4%), temperate sub-continental (28.6%); the sum of areas equipped for irrigation in the oceanic-temperate, continental temperate, boreal and arctic zones are less than 5%. Since 85.5% of total harvested areas are in the northern hemisphere, in temperate regions January is mostly a winter month: this explains the substantial negative differences found where the HS method is less effective due to low temperatures. On the other hand, HS and PM methods are more aligned on tropical (orange) and sub-tropical regions (beige and yellow). Also the temperate sub-continental region (green), that accounts the largest fraction of temperate areas, shows a good alignment during summer and winter. In summary, except for a few isolated cases on tropical and temperate-continental regions, we found monthly correlation indices between HS and PM typically higher than 0.8. This is consistent with results from previous studies, where HS was found a reliable method on a global scale [28] and also with studies in arid and semi-arid regions where R reaches 0.97 [29].

Although ERA5 provides data of potential evapotranspiration, *ET*_*p*_ (defined as the maximum amount of evaporation, under existing atmospheric conditions, from a surface of pure water), this variable is the result of an energy-balance approach [30] which is conceptually different from *ET*_*0*_. We have compared our results with this dataset, finding marked local differences, with annual rate of *ET*_*0*_ computed with HS being up to 25% lower than *ET*_*p*_, with winter *ET*_*p*_ being too large in central Asia and North America and with unclear low values in some intensively harvested regions (central France, the Amazon, the Great Lakes region of the USA, Myanmar, Laos, New Guinea). For these reasons, *ET*_*p*_ is not recommended to be used in the assessment of crop water requirements.

## 3. Methods

For assessing irrigation requirements on a global scale, a water balance model has been developed, improving the methodology proposed by Tuninetti et al. [20]. The model calculates the irrigation requirement using a soil-water balance on land equipped for irrigation, taking as input the climatic data and the agricultural information described in the previous paragraphs. We evaluated the actual evapotranspiration (*ET*_*a*_) for each day, according to the FAO’s approach [18], i.e.

$$ET_{a,i}=ET_{0,i}\cdot k_{c,i}\cdot k_{s,i},$$ (2)

where *ET*_*0*_ and *ET*_*a*_ are expressed in mm/day, *i* is the specific day, *k*_*c*_ is a dimensionless coefficient specific for each crop and growing phase (or crop coefficient), and *k*_*s*_ is the water stress coefficient, that takes values from 0 to 1.

When *k*_*s*_ = 1, the evapotranspiration is not affected by water stress and reaches the maximum rate, named crop evapotranspiration (*ET*_*c*_). If *k*_*s*_ = 0, the crop reaches the wilting point because of the dry soil condition, and there is no evapotranspiration.

The study of Chapagain & Hoekstra [21] provides the crop-specific information to divide the growing period into four phases (initial, crop development, mid-season, late season) and assigning daily crop coefficients. As described by Allen et al. [18], the crop coefficient remains constant during the initial stage (*k*_*c*_ = *k*_*c*,*ini*_). In the development stage, *k*_*c*_ grows linearly from *k*_*c*,*ini*_ to *k*_*c*,*mid*_, i.e. the constant value of the coefficient in the mid-season stage, and finally, in the late season stage, it decreases linearly from *k*_*c*,*mid*_ to *k*_*c*,*end*_. This information is available for the ten different climatic zones summarized in Fig 2 (based on the GAEZ thermal agro-classification [22]).

The water stress coefficient *k*_*s*_ is calculated according to the FAO methodology [18], i.e.

$$k_{s,i}=\left\{\begin{array}{ll} 1 & \text{if}\cdot S_{i}\geq \left(1-\rho_{i}\right)S_{fc}\\ \frac{S_{i}-S_{w}}{\left(1-\rho_{i}\right)\left(S_{fc}-S_{w}\right)} & \text{if}\cdot S_{w}<S_{i}<\left(1-\rho_{i}\right)\left(S_{fc}-S_{w}\right)\\ 0 & \text{if}\cdot S_{i}\leq S_{w} \end{array}\right.$$ (3)

In Eq (3), *S*_*i*_ is the soil moisture [mm] in day *i*, calculated multiplying the water content [%] and depth of the root zone [mm]. In the same equation, *S*_*fc*_ and *S*_*w*_ are the levels of soil moisture [mm] corresponding to field capacity (i.e. the maximum amount of water that can be stored in soil after drainage by gravity action) and wilting point (i.e. the dry soil condition, when crops do not have available water), respectively. The depletion fraction, *ρ*_*i*_, is the percentage of total available water that can be used by a crop without reaching water stress. The term (1 –*ρ*_*i*_)*S*_*fc*_, also known as *S*_i_*, is the soil moisture at incipient water stress or stomata closure [mm]. According to the FAO methodology [18], the depletion fraction *ρ* of day *i* can be calculated as

$$\rho_{i}=\rho_{st}+0.04\left(5-ET_{c,i}\right),$$ (4)

where *ET*_*c*,*i*_ is the crop evapotranspiration in the absence of water stress [mm] and *ρ*_*st*_ is a crop-specific standard value of the depletion fraction at *ET*_*c*_ = 5mm/day. This expression is related to the sensitivity of crops to weather conditions, as higher temperatures imply faster stomata closure, in equal soil moisture conditions. The depletion fraction is found to vary in a range between 0.1 and 0.8.

To apply Eq (3), we used the 30-arc-sec global dataset of available water capacity, i.e. the difference between field capacity and wilting point, from the Harmonized World Soil Database by JRC [31].

We used the data from Allen et al. [18] to set the maximum rooting depths for irrigated lands: roots of temporary crops are supposed to increase linearly in the first two phases of the growing period, from a sowing depth of 0.2 m, and then remain equal to the maximum value for the rest of the season. Roots of perennial crops are supposed equal to the maximum length for the entire year. In this way, the model calculates the actual available water for each day of the growing period.

The daily soil-water balance expresses the variation of soil moisture in the root zone, calculated as a function of inputs and outputs:

$$S_{i+1}-S_{i}=P_{i}+I_{i}-ET_{a,i}-PS_{i},$$ (5)

In Eq (5), all variables are expressed in mm. *S*_*i*_ is the soil moisture on day *i* and ranges between field capacity and wilting point conditions (*S*_*w*_ ≤ *S*_*i*_ ≤ *S*_*fc*_); *S*_*i+1*_ is the soil moisture resulting from the daily water balance, used as the initial condition on the following day; *P*_*i*_ is precipitation; and *ET*_*a*,*i*_ is the actual evapotranspiration, calculated according to Eq (2). During dry periods, low rainfall may be insufficient to compensate for evapotranspiration and soil moisture reaches the water stress level (S*), the condition in which plants start to close their stomata. The daily irrigation requirement, *I*_*i*_, is defined as the water needed to avoid water stress, the additional depth that guarantees *S*_*i*_ ≥ (1 –*ρ*_*i*_)*S*_*fc*_ according to Eq (3). In the event that daily precipitation brings soil moisture to field capacity, any further input of rainfall that cannot be stored in the soil is called precipitation surplus, *PS*_*i*_. In Eq (5), this variable represents the sum of runoff and ground percolation. Since irrigated fields are usually almost horizontal, surface runoff and groundwater lateral movements in the root zone were considered negligible: *PS*_*i*_ is assumed equivalent to deep percolation.

On the sowing date of temporary crops, the initial soil moisture is assumed equal to field capacity. For crops grown on paddy fields a specific hypothesis is required: an additional depth of 200 mm is considered to saturate the soil before each sowing date, as suggested by the FAO methodology [32]. This water is not included in the estimation of rice irrigation requirement, because it is not directly used by the plants, and is explicitly reported in the results.

Considering that precipitation may occur in different hours of the day, even during the night when evapotranspiration is negligible, we introduced a random assignment of input rainfall, occurring before or after the calculation of *ET*_*a*_.

The model evaluates the irrigation requirement for each growing season in a cell. If a crop is repeatedly cultivated on the same field, the final amount of *I* is the sum made for all seasons. Instead, if the same crop is cultivated on different fields within the same cell, the final *I* for that crop is the area-weighted average between the two requirements. The total volume of irrigation requirement is calculated on a monthly or annual scale, considering the contribution of the 26 crops, as

$$V=10\overset{26}{\underset{c=1}{\sum }}\left(I_{c}\cdot AEI_{c}\right),$$ (6)

where *V*, *I*_*c*_ and *AEI*_*c*_ are, respectively, the total irrigation requirement volume [m^3^], the irrigation requirement [mm] and the irrigated area [ha] for a specific crop *c*.

The averaged requirement considering the entire area of a cell is *I*_*grid*_ = *V*/(10 *A*_*grid*_), computed as the ratio between the total volume [m^3^] of irrigation requirement and the area *A*_*grid*_ [ha] of the 0.0833° grid cell: this is used here to compare irrigation requirements from different regions in the world. The spatial variability of *I*_*grid*_ values is consistent with the actual distribution of irrigated areas and cropping intensities within cells.

## 4. Results

### 4.1. Global irrigation requirement

**Fig 3:**
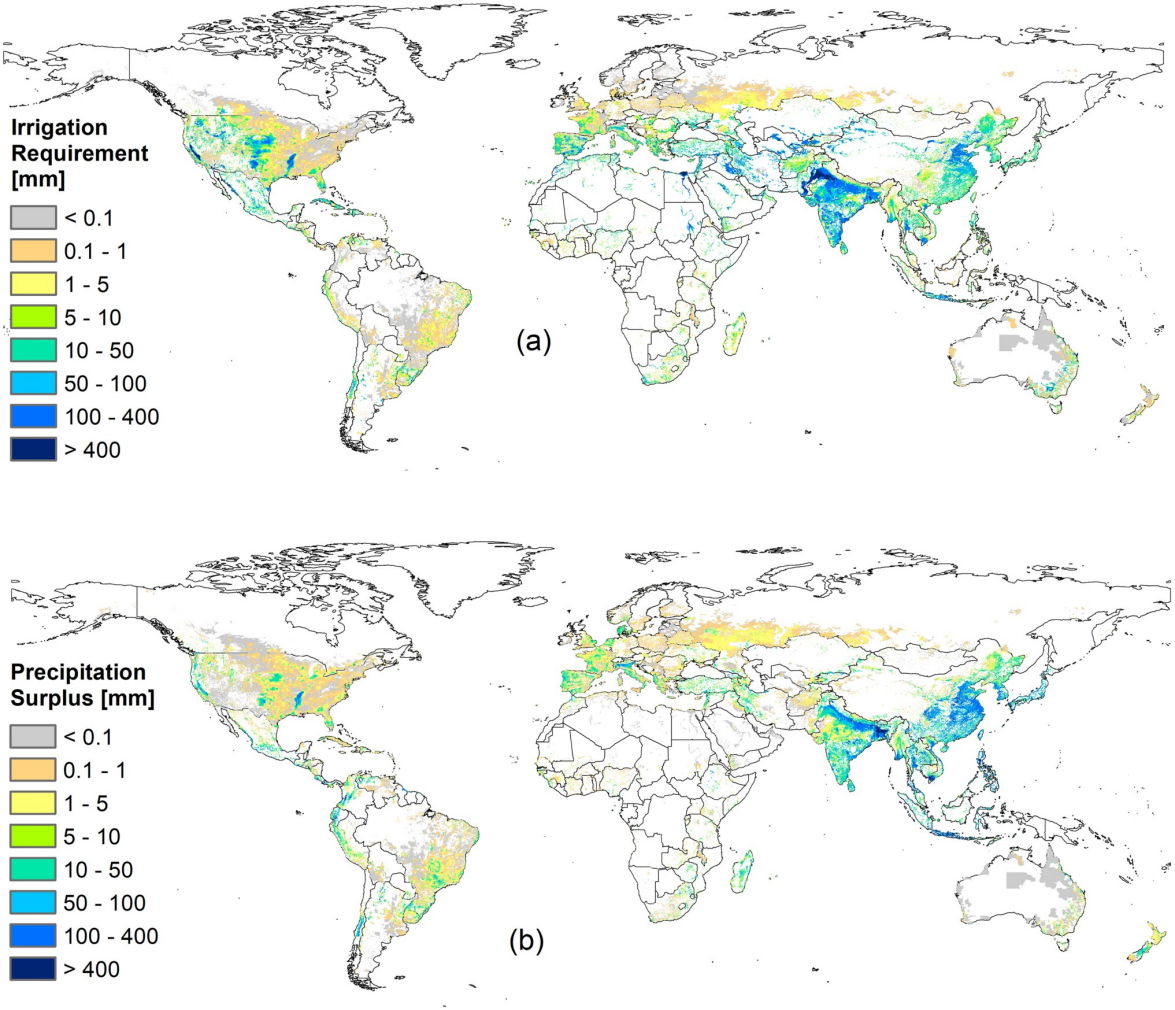
Spatial distribution of irrigation requirement and precipitation surplus. Cell-average water depths [mm], as a ratio between the total volume cumulated over the year within the cell and the cell area.

The model described above was applied using the climatic and agricultural data of year 2000, and the global volume of irrigation requirement was estimated in 962 km^3^. Fig 3a shows the spatial distribution of annual cell-averaged requirements (*I*_*grid*_) and Fig 3b also shows the spatial distribution of cell-averaged precipitation surplus (*PS*).

Factors influencing the *I*_*grid*_ are the ratio between annual precipitation and the reference evapotranspiration (i.e. the Budyko index), the temporal variability of these forcings (even in wet regions a significant amount of irrigation may be required if the precipitation is strictly seasonal), and the extension of AEI in each pixel of the grid. For example, in Bangladesh the Budyko index is 1.5 but the high rainfall rate is concentrated in the monsoon season, and the tropical high evapotranspiration quickly leads crops to water stress during dry periods: so we found high *I*_*grid*_. In the central and northern parts of Australia, the irrigation requirement is typically higher than 900 mm/year, but the very low density of harvested areas keeps the cell average *I*_*grid*_ below 1 mm/year (in this region, less than 0.1% of the territory is usually equipped for irrigation). In arid and semi-arid regions we found high *I*_*grid*_ values (e.g. over 750 mm/year in the Indo basin, over 800 mm/year in the Nile delta and over 720 mm/year in California) and negligible precipitation surplus due to the lack of precipitation. In many European and American temperate regions, high *I*_*grid*_ levels usually depend more on *AEI* density than on climate conditions. For instance, Italy’s northern Po Valley requires much more irrigation than southern territories: despite south of Italy is typically drier, northern high rates depend on the density of harvested areas (especially rice paddies).

The global precipitation surplus (*PS*) over irrigated lands was found as 672 km^3^ and the spatial distribution of *PS*_*grid*_ is shown in Fig 3b. Although *PS* is a “water loss” for the water balance of the root zone, it has an important role in the hydrological cycle and ecosystem functioning. This rainfall water is not used by the crops, but it is an important source of recharge for aquifers.

The comparison of national estimates (i.e. the sum of *I* and *PS* volumes from Eq (6) over national areas) shows that 171 countries in the world require irrigation, and in 106 of them the *PS* volume is higher than *I*. If part of this precipitation surplus could be stored and used during the growing season, this would lead to a significant decrease in water withdrawals. For example, the irrigation required by China was estimated to the second highest in the world (130 km^3^/year) and even a higher precipitation surplus (193 km^3^/year); in theory, it could be possible to satisfy all the Chinese irrigation requirements by using annual precipitation on *AEI*.

Despite the apparent similarity between Fig 3a and 3b, we found a low annual correlation between irrigation requirement and precipitation surplus: the Pearson index (R) between *I*_*grid*_ and *PS*_*grid*_ is 0.38. We found an even lower correlation comparing the annual distribution of *I* and *PS* (water depths over *AEI*) for which *R* = −0.16: the higher correlation between *I*_*grid*_ and *PS*_*grid*_ is due to their common dependence on irrigated areas and cropping intensities. On a monthly scale, where the variability of *P* and *ET*_*0*_ is more important, we found higher correlations between *I* and *PS* (e.g. R_July_ = -0.29 and R_December_ = -0.20).

In considering individual crops it is significant that, according to FAOSTAT, more than 48% of total agricultural production in tonnes in year 2000 included only four crops: sugar cane, rice, wheat and maize. The irrigation required by these four was 58% of the global estimation. Rice’s requirement is the largest, nearly 30% of total. This is mainly because of the huge extension of paddies, the high cropping intensity (e.g. in Uttar Pradesh, Haryana and Punjab in northern India, there are three consecutive growing seasons of rice per year) and the high sensitivity of rice to soil moisture depletion. Paddies are artificially maintained at a high moisture level, and field capacity is frequently reached even with weak precipitation, with a consequent major loss of *PS* during rainfall events in the growing period. Table 2 summarizes our findings on the crop-specific results, classifying them by the extent of irrigated areas from MIRCA2000.

**Table 2:** Crop-related summary of estimations and irrigated areas.

CROP	I [km^3^]	PS [km^3^]	AEI [10^6^ ha]	CI
Rice	271.4	360.1	64.0	1.6
Wheat	153.1	44.3	59.3	1.1
Maize	66.0	48.7	29.9	1.0
Others annual	51.8	19.8	16.7	1.2
Cotton	73.3	21.2	16.3	1.0
Others perennial	80.0	48.5	12.9	1.0
Fodder grasses	71.8	14.5	11.7	1.0
Sugar cane	71.7	50.9	10.2	1.0
Soybean	16.6	15.3	6.0	1.0
Pulses	18.0	2.8	5.5	1.0
Barley	7.3	4.1	4.6	1.0
Potato	12.4	5.8	3.7	1.0
Groundnuts	7.5	9.6	3.7	1.0
Citrus	15.1	12.2	3.6	1.0
Rape seed	6.7	0.5	3.4	1.0
Sorghum	8.0	3.4	3.4	1.0
Millet	3.5	1.9	1.7	1.0
Grapes	7.4	4.0	1.7	1.0
Sugar beets	6.1	0.89	1.6	1.0
Sunflower	3.6	1.8	1.3	1.0
Date palm	8.4	0.14	0.7	1.0
Rye	0.74	0.68	0.4	1.0
Coffee	0.67	0.83	0.2	1.0
Cocoa	0.01	0.06	0.01	1.0
Cassava	0.02	0.03	0.01	1.0
Oil palm	0.04	0.11	0.01	1.0

I: Irrigation requirement; PS: Precipitation surplus; AEI: Areas equipped for irrigation; CI: **C**ropping intensity, i.e. the number of yearly growing seasons per field (CI>1 for crops harvested more than once per year on the same fields, like rice in northern India). Rice’s requirement does not include additional amounts of water to saturate the fields before sowing (globally 206.2 km^3^).

**Fig 4:**
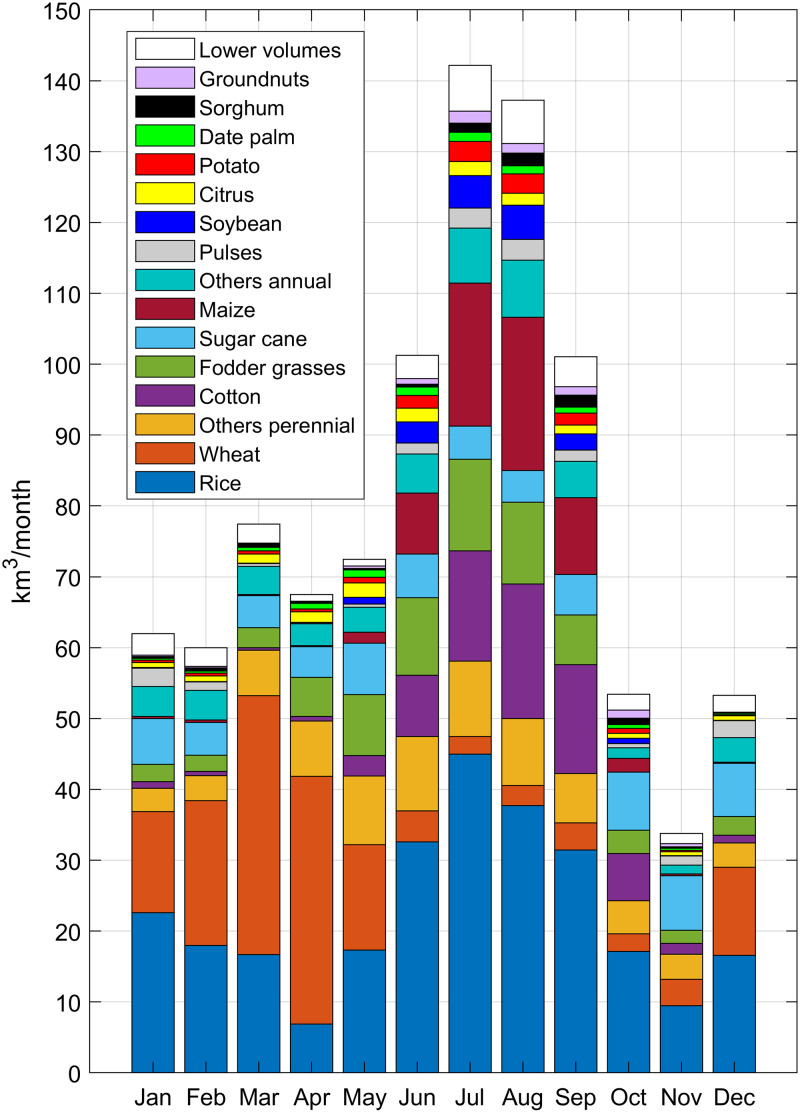
Monthly volumes of crop-specific irrigation requirement [km^3^/month]. Rice volumes refer only to the evapotranspiration requirement.

Fig 4 shows the temporal distribution of crop-specific volumes of irrigation requirements for year 2000, calculated according to Eq (6) and cumulated at monthly scale. The largest volume is required from June to September, mainly because most of the summer crops are harvested in the northern hemisphere (e.g. 90% of maize *I* is required during these four months). In contrast with other seasonal crops, wheat is massively cultivated during winter and spring, and requires more irrigation from December to May. For example, more than 46% of wheat *AEI* are in India and Pakistan, where this crop is mainly harvested from November to May and most of the rainfall occurs in the summer. Winter wheat is also largely harvested in Europe, U.S. and China, and is usually planted between October and December. In contrast, in the northern part of India (e.g. Punjab) wheat is mainly harvested from June to November as well as in southern Europe and in the US Northwest. The monthly volumes of irrigation required by rice, wheat and cotton are well aligned with results from the GCWM model, described by Siebert & Döll [9], even if we found lower values of total irrigation requirement in spring months.

### 4.2. Regional results on three intensive areas

**Fig 5:**
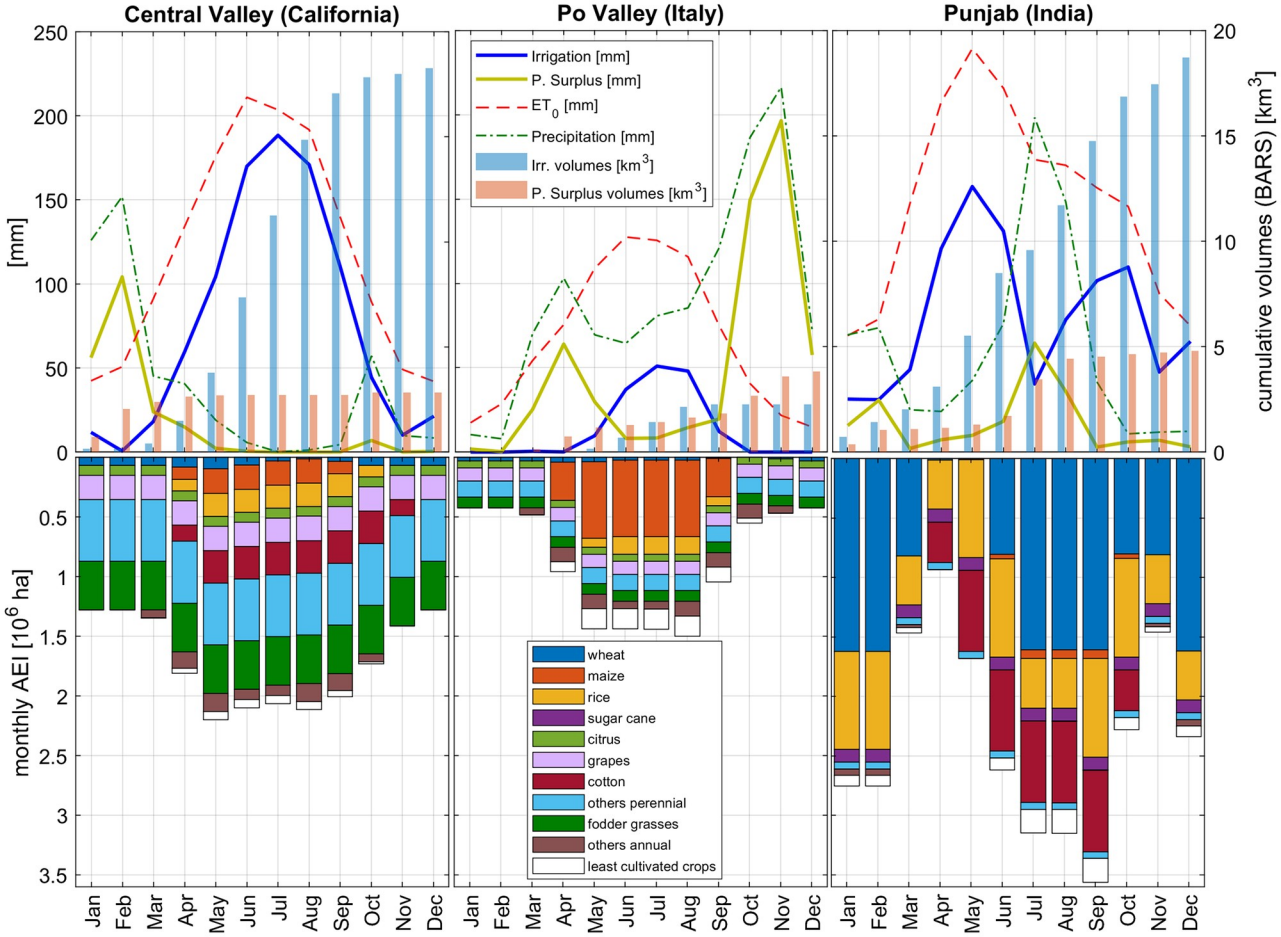
Monthly variability of climate forcings and model results, over three intensively harvested areas. The selected regions belong to different agro-climatic zones: Central Valley (California, sub-tropic winter rainfall), Po Valley (Italy, temperate sub-continental) and Punjab (India, sub-tropic summer rainfall). The variables on both the axes have been quantified considering the monthly harvested *AEI*, as shown in the lowest part of the plots.

To better explore the spatial variability of results and to verify the ability of the model to provide estimates for various climate scenarios, the monthly water balance on three intensively harvested regions has been analyzed in more detail. These are: Central Valley (CV, California), Po Valley (PV, Italy) and Punjab (PU, India), belonging respectively to sub-tropic winter rainfall, temperate sub-continental and sub-tropic winter rainfall climatic zones. The three areas have similar geographical extents (about 52000 km^2^, 47000 km^2^ and 50000 km^2^ respectively), but heterogeneous portions of areas equipped for irrigation (42%, 32% and 72% respectively). Fig 5 shows the monthly variability of the main water-balance terms, i.e. the climate variables (*P* and *ET*_*0*_), the area-weighted values of irrigation requirements, and precipitation surplus. The cumulative volumes of *I* and *PS* are also shown (on the right axes). In the lower part of each panel, the bar graphs show the monthly distribution of AEI by actual crop harvested. CV is mainly cultivated with perennial crops (23% of total *AEI*), fodder grasses (18%), grapes (9%) and cotton (12%); PV has maize (43%) and rice (10%) in the warm season and perennial crops over the entire year (9%); PU is mainly harvested with wheat (45%), rice (23%) and cotton (19%).

Irrigation requirements of CV and PU are comparable (19.0 km^3^ and 20.6 km^3^ respectively) but monthly values are very different, mainly because of the difference in precipitation and evapotranspiration rates. In California, the maximum irrigation requirement is close to 190 mm for the month of July due to the combination of high *ET*_*0*_ and very low precipitation. In this region, all the water surplus is concentrated in the winter months, with a maximum of 105 mm in February, while the high evapotranspiration rates of warm months (e.g. over 150 mm/month in all the summer period) maximize the irrigation requirement. The Po Valley average reference evapotranspiration is lower and precipitation is higher than in CV and PU, due to the temperate climate. *I* reaches 50 mm only in July: a short period if compared with five months in California and six in Punjab. Almost no irrigation is required in Po Valley from October to April, due to the combined effect of high rainfall and low *ET*_*0*_. In this area, the irrigation requirement is mainly concentrated in the summer period, but it only reaches 2.2 km^3^ (a small volume, compared with CV and PU). Annual water surplus over PV is 3.8 km^3^, a huge volume if compared with the two other regions, but this surplus is mainly concentrated in the October-November period. In fact, in year 2000 Po Valley was afflicted by an intense flood event in November, so we can assume the high water surplus is strongly related to that specific event.

### 4.3. Classification over water-stressed areas

Additional insights from the results can be gained by grouping the volumes of irrigation requirement according to the classes of agricultural water risk described in the Water Risk Framework, published by the Water Resources Institute [33]. This framework includes a global dataset of water risk indices (referring to the 1960–2014 period), combining indicators of physical risk, water quality and regulatory aspects for several human activities. We used the “Agricultural Water Risks” indices to classify the irrigation requirements assessed by our model.

**Fig 6:**
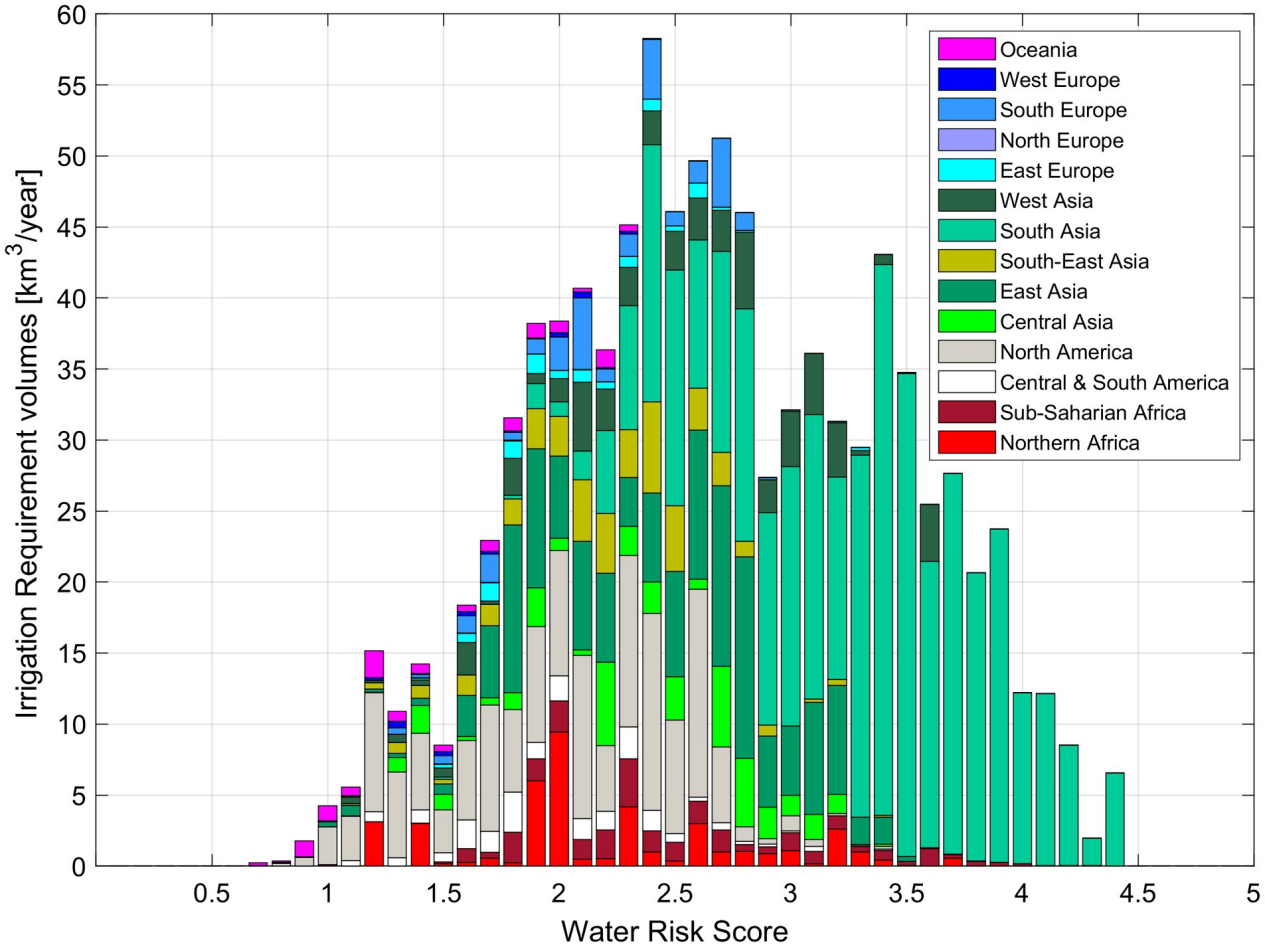
Distribution of irrigation requirement volumes by classes of agricultural water risk. Classification of the World Resources Institute: Low risk (0–1), Low-Medium risk (1–2), Medium-High risk (2–3), High risk (3–4), Extremely High risk (4–5).

Fig 6 shows the distribution of irrigation requirement volumes by classes of agricultural water risk: a high risk means that the requirement volume may be hard to satisfy. Irrigation water could be unavailable (quantitative risk) or polluted (qualitative risk), and this may be critical for crops.

Only 1.2% of global *I* volume falls within the low-risk class, mainly required in North America and Oceania. Unfortunately, 44.5% of irrigation requirement is exposed to a medium-high risk, and 28.4% to a high or extremely high risk.

Southern Asia is the most exposed area to agricultural water risk: most of the irrigation required in this region (about 25% of global *I*) falls into the medium-high, high and extremely high-risk classes. This is due to the fact that India and Pakistan are two of the most productive countries, with two of the most water requiring agricultural systems.

About 87% of North American *I* is affected by low and medium risk, with western states (e.g. California) being more exposed to quantity and quality limitations than are the central and eastern regions. In North Africa, agricultural areas along big rivers are less exposed by risk (e.g. the Nile delta) while the small fraction of croplands in the arid and semi-arid territory is exposed to medium or high risk. The largest part of European irrigation requirement is exposed to medium risk, but the nature of the risk is different depending on the climate region (e.g. Po Valley is more exposed to quality-related risk, while Spain and southern Italy to quantity-related risk).

## 5. Discussion

### 5.1. Comparison with other global assessments

In comparing the results of this study with previous assessments, one must take into account that all global assessments are affected by some uncertainty. The quality of input data, spatial and temporal resolution, modelling assumptions such as the length and number of growing seasons or the classification of some crops in macro-categories (e.g. “others annual”) play an important role in the assessment of irrigation requirements. In this study, a part of the uncertainty is reduced using a model based on actual daily data of precipitation and temperature.

Table 3 shows a comparison of irrigation requirement results from the global models introduced in Table 1. The comparison points out the alignment of our estimation to the literature and how much the improvement of the input detail affects the final result.

**Table 3:** Comparison of global estimations of irrigation requirement.

Assessment		I [km^3^/year]
This study		962
[12]	Chiarelli et al. (2020)	1068
[11]	Mekonnen & Hoekstra (2011)	899
[10]	Liu & Yang (2010)	927
[9]	Siebert & Döll (2010)	1180 ^(i)^, 1448^(ii)^, 1145^(iii)^
[8]	Hanasaki et al. (2008)	1320
[7]	Rost et al. (2008)	1364
[6]	De Fraiture (2007)	1450
[5]	Döll et al. (2002)	1091.5

^(i)^ Estimates based on Penman-Monteith reference evapotranspiration.

^(ii)^ and ^(iii)^ Estimates from two approaches based on Priestley-Taylor reference evapotranspiration.

The estimate from this study is well aligned with most of the previous works, especially with assessments based on the MIRCA2000 dataset. The differences between the latest models may have several causes: the use of different climate datasets, the modelling approach for the crop growth (e.g. Siebert & Döll [9] use average global values of crop coefficients, neglecting climate-related differences) and the initial soil moisture conditions (e.g. Chiarelli et al. [12] performed a sensitivity analysis assuming three different scenarios to simulate the initial moisture condition on the sowing date). The GCWM model [9] from Siebert & Döll provides three results, obtained using different methods to estimate the reference evapotranspiration: 1180 km^3^/year using Penman-Monteith and two results using different alternatives of the Priestley-Taylor method (1448 km^3^/year and 1145 km^3^/year). The estimate from the H07 [8] model (1320 km^3^/year) was reported by Siebert & Döll [9], and refers to the 1986–1995 period.

Compared to later studies, the older models seem to overestimate the irrigation requirement. This is probably due to the fact that most of these models were not based on crop-specific data of irrigated areas and growing calendars, so the assessments were performed with a larger number of assumptions, using average values to model the crop development. For example, Döll et al. (2002) performed the assessment classifying rice paddies separately and aggregating all the other crops [5].

### 5.2. Comparison with national data

Model results, in terms of irrigation requirement volumes, have been cumulated at the national scale and compared with data of agricultural withdrawal (*W*) for the year 2000, provided by AQUASTAT [32] and by the Organization for Economic Co-operation and Development (OECD) [34]. The national irrigation withdrawals are the volumes of water taken from surface water bodies or groundwater to be used in agriculture, in fields equipped for irrigation. Withdrawals include the volumes required by crops and the water losses due to inefficiencies in the distribution and irrigation systems.

The ratio between *I* and *W* can be reasonably associated with a national mean irrigation efficiency (*E*), defined as the ratio between the amount of water withdrawn for agricultural purposes and the theoretical volume required by crops. The irrigation requirements are equal to withdrawals in the ideal condition of maximum efficiency (no water losses in the irrigation system) and absence of water stress during the growing season. In the real systems withdrawals are generally higher than requirements. The comparison between these two volumes is important to validate our estimations: the difference between *I* and *W* may depend on the technological level of the country, on the harvested crops (e.g. the irrigation efficiency of rice paddies is generally very low) and on the availability of freshwater.

**Fig 7:**
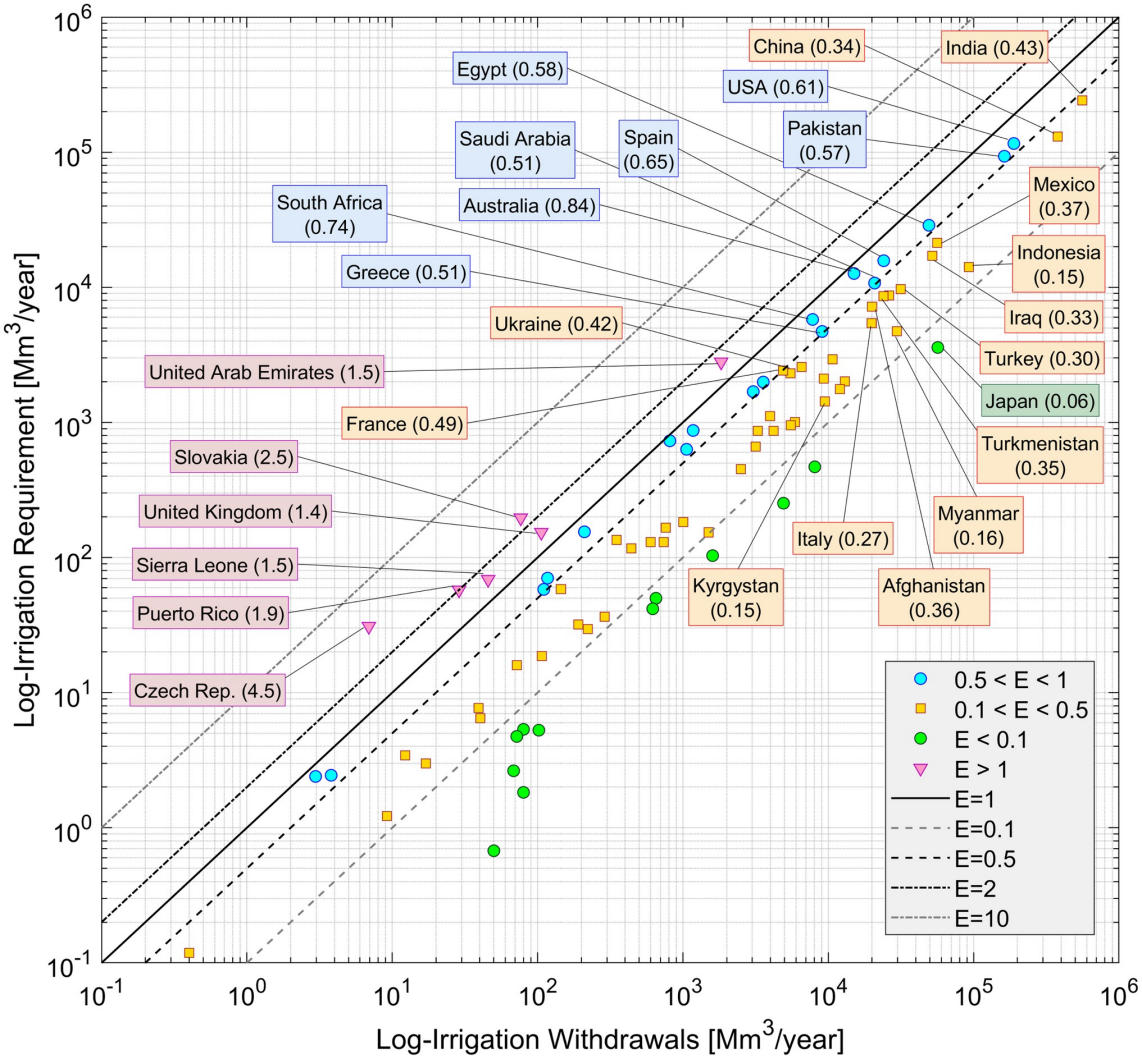
Comparison of national irrigation requirements and withdrawals and aggregation by classes of national efficiency. Blue circles, red crosses and green squares are countries where *I*<*W*, grouped by classes of *E*. Purple triangles are countries for which the model estimates *I* higher that actual *W*. The bold continuous line indicated the condition of *I* = *W*; classes of efficiencies lower than 1 are limited by dashed lines, while the two dotted lines delimit classes of efficiencies higher than 1. For E<1, the labeled countries have at least 10^6^ ha of areas equipped for irrigation.

Fig 7 shows the logarithmic scatter plot of the 81 national withdrawals and requirement volumes. Nations are grouped by classes of efficiency, delimited by dotted, dashed and continuous lines: 45 nations lie between 0.1 and 0.5 and 18 nations between 0.5 and 1. More than 80% of required volumes belong to nations within these ranges (54% belongs to nations with efficiency between 0.1 and 0.5). India, China and the USA are the countries with higher irrigation requirement: 25.4%, 13.5% and 12.0% of global irrigation volumes, *I*, respectively. Spain is the European country with the highest irrigation requirement (15.7 km^3^/year, near 1.6% of global *I*).

For 12 countries, the irrigation efficiencies were estimated to be lower than 0.1: in most of these countries the irrigation water volumes are very small, due to the lack of irrigated lands. Japan appears to have a surprisingly low efficiency (0.06), considering its economic and technological levels, but this may be a consequence of a massive presence of rice paddies (about 54% of total harvested areas, according to FAOSTAT) which have a very low irrigation efficiency: Döll et al. [5] assume an irrigation efficiency for rice lower than 0.1.

For 6 nations the mean efficiency is higher than 1 (irrigation requirement higher than agricultural withdrawals), e.g. United Kingdom (1.4), United Arab Emirates (1.5), Czech Republic (4.5). Withdrawals lower than requirements in nations with high water-demand states like United Arab Emirates may be due to deficit irrigation practices, in which a provision of lower water volumes than actual requirements are due to lack of water availability [35]. Results are consistent with the efficiencies estimated by Siebert & Döll [36], with similarities in some critical nations (e.g. they obtain E = 2.55 in Czech Rep. and E = 1.79 in UK). In some of these nations, high efficiency values may be due to the low magnitude of irrigation requirement and withdrawals.

### 5.3. U.S. irrigation requirements

A more detailed validation of the model has been done using data from the USA, for which the Geological Survey (USGS) provides local information about irrigation withdrawals in year 2000 [37]. Irrigated lands are more concentrated in the western US, especially in the Central Valley of California, in Idaho and other northwestern states, but also in Nebraska and Arkansas (concentrated in the Mississippi region). According to this dataset, the USA withdrawal for irrigation was 196.4 km^3^ in year 2000. Building a weighted linear regression between the observed withdrawals and the irrigation requirements in each USA state (using *AEI*, i.e. Areas Equipped for Irrigation, as weight), we estimate an angular coefficient (i.e. the expression of the overall irrigation efficiency) of 0.58. This result is comparable with the value of 0.6 provided by Döll et al. with the same procedure [5].

**Fig 8:**
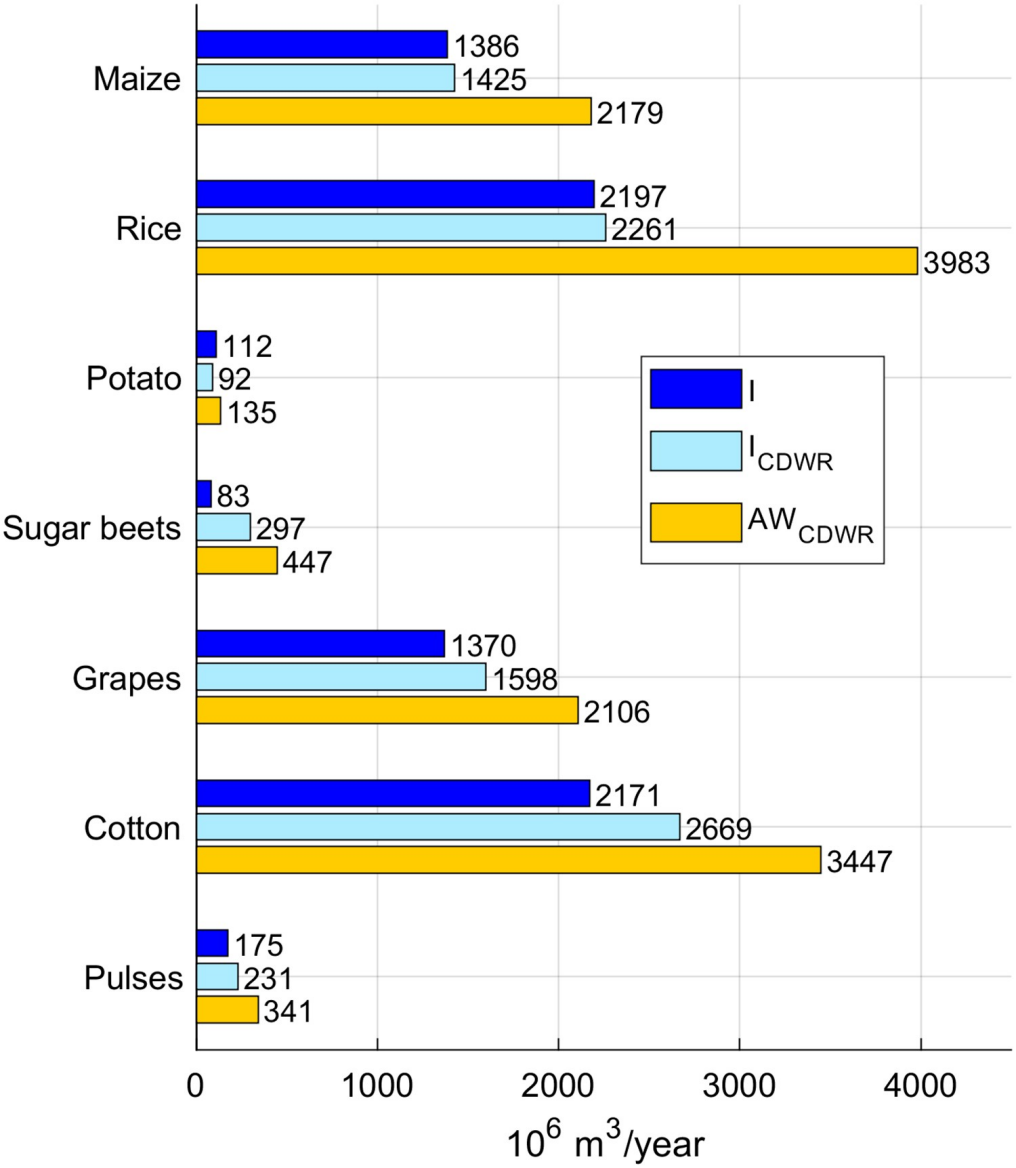
State of California: Comparison between crop-specific irrigation requirement estimations (*I*), data of applied water (*AW*_*CDWR*_) and evapotranspired applied water (*I*_*CDWR*_). The *AW* and *I*_*CDWR*_ data are from the “California Department of Water Resources”, referring to year 2000.

California is the US state with the largest extension of *AEI* and presents an irrigation efficiency of 0.64 (higher than the average value of United States). Fig 8 shows the comparison between our crop-specific estimates of irrigation requirement (*I*) and the data of applied irrigation water (*AW*), which is the volume of withdrawn water that is actually delivered to the crop fields. This information is available for seven crops, provided by the California Department of Water Resources (CDWR) for year 2000 [38]. Rice is the crop with the largest difference between *I* and *AW*, and this is consistent with the low efficiency associated to paddies. The same dataset also provides estimates of the crop-specific irrigation requirements in California. We validated our model comparing our results with the estimates from the CDWR (*I*_*CDWR*_), as shown in Fig 8.

The irrigation requirements obtained in this work are well aligned with the *I*_*CDWR*_: for example, we find results for maize, rice and grapes corresponding to 98.0%, 94.5% and 93.7% of *I*_*CDWR*_ estimations respectively. Large differences between *I* and *I*_*CDWR*_ volumes for cotton and sugar beets are consistent with the differences of cropland extent between CDWR and MIRCA2000. The ratio between *AEI* from MIRCA2000 and CDWR is 0.27 for sugar beets and 0.73 for cotton, and the ratios between *I* and *I*_*CDWR*_ are 0.29 and 0.85 respectively.

Unfortunately, the other crop-specific information available in California are grouped into categories (e.g. fruits or vegetables) that do not match the MIRCA2000 dataset, thus further comparisons cannot be performed.

## 6. Conclusions

This work presents a model for the assessment of global irrigation requirement based on the high-resolution dataset ERA5 from the Copernicus Climate Data Store. The model assesses the minimum irrigation required to avoid water stress, working on a daily soil water balance and modelling the crop development according to the FAO methodology, limiting uncertainties related to climate-forcing data by using satellite information. The reference evapotranspiration is calculated following the Hargreaves-Samani method, which requires only information about surface temperature and solar radiation, and fits very well when compared to more complex and data-intensive methods like Penman-Monteith.

The model was used to assess the irrigation requirements for 26 crops in the world, working on crop-specific agricultural areas equipped for irrigation, while the focus on year 2000 is motivated by agricultural data availability permitting comparison of results with previous studies, many of which were focused on that year. Due to the difficulties in simulating crop development on a global scale in many different climatic, technological and cultural scenarios, the main uncertainty remains the correct modelling of the length of growing periods.

The global irrigation requirement for year 2000 was found to be about 962 km^3^, which is an amount comparable both with results from models driven by long-term climate data and from models working with monthly time series. The spatial distribution of irrigation requirements (0.0833°) points out their dependency on the extension of local areas equipped for irrigation, the crop intensity and the kind of crop harvested, as well as the lack of precipitation during growing periods. The model also estimates that an important amount of rainfall occurring along the growing seasons is lost as surplus over irrigated lands (672 km^3^, about 68% of irrigation requirement), mainly motivated by the seasonal variability of precipitation.

The comparison between irrigation requirements and national data of agricultural water withdrawals shows a good agreement between the two variables, with reasonable values of irrigation efficiencies. The model has been validated through a comparison between crop-specific estimations of irrigation requirements and data provided by the California Department of Water Resources, which highlights a very good fitting.

The classification of irrigation requirements by classes of agricultural water risk shows that most of the requirement in South and East Asia is exposed to high and very high risk of not being satisfied, due to possible unavailability of irrigation water in terms of quantity and quality. The global estimation described in this work is a first step of a wider project of irrigation assessment, in which the temporal variability and the use of additional data from remote sensing are foreseen.

## Supporting information

S1 File (RAR)
